# Multiscale landscape genomic models to detect signatures of selection in the alpine plant *Biscutella laevigata*

**DOI:** 10.1002/ece3.3778

**Published:** 2018-01-10

**Authors:** Kevin Leempoel, Christian Parisod, Céline Geiser, Stéphane Joost

**Affiliations:** ^1^ Laboratory of Geographic Information Systems (LASIG) School of Civil and Environmental Engineering (ENAC) École Polytechnique Fédérale de Lausanne (EPFL) Lausanne Switzerland; ^2^ Laboratory of Evolutionary Botany University of Neuchâtel Neuchâtel Switzerland; ^3^ Institute of Plant Sciences University of Bern Bern Switzerland

**Keywords:** amplified fragment length polymorphism, digital elevation model, landscape genomics, local adaptation, multiscale analysis

## Abstract

Plant species are known to adapt locally to their environment, particularly in mountainous areas where conditions can vary drastically over short distances. The climate of such landscapes being largely influenced by topography, using fine‐scale models to evaluate environmental heterogeneity may help detecting adaptation to micro‐habitats. Here, we applied a multiscale landscape genomic approach to detect evidence of local adaptation in the alpine plant *Biscutella laevigata*. The two gene pools identified, experiencing limited gene flow along a 1‐km ridge, were different in regard to several habitat features derived from a very high resolution (VHR) digital elevation model (DEM). A correlative approach detected signatures of selection along environmental gradients such as altitude, wind exposure, and solar radiation, indicating adaptive pressures likely driven by fine‐scale topography. Using a large panel of DEM‐derived variables as ecologically relevant proxies, our results highlighted the critical role of spatial resolution. These high‐resolution multiscale variables indeed indicate that the robustness of associations between genetic loci and environmental features depends on spatial parameters that are poorly documented. We argue that the scale issue is critical in landscape genomics and that multiscale ecological variables are key to improve our understanding of local adaptation in highly heterogeneous landscapes.

## 1 INTRODUCTION

Sessile plants have been shown to locally adapt to their environment (Linhart & Grant, 1996). The high heterogeneity of environmental conditions being considered as the principal trigger of local adaptation in face of homogenizing gene flow, fine‐scale genetic differentiation has commonly been interpreted as a result of strong selection pressures across natural landscapes (Gonzalo‐Turpin & Hazard, 2009; Gray et al., 2014; Parisod & Christin, 2008; Vekemans & Hardy, 2004). Preponderant abiotic factors driving such adaptation have, however, rarely been identified, mainly because the ecological conditions acting on individual plants are difficult to characterize.

Mountainous areas are ideal to study high genetic differentiation and local adaptation at a fine scale (Parisod & Bonvin, 2008; Stöcklin, Kuss, & Pluess, 2009). These habitats are indeed highly heterogeneous, and topography plays a considerable role in local climatic variability (Wilson & Gallant, 2000). Until recently, existing climatic datasets were, however, too coarse to account for environmental heterogeneity at fine scales. Furthermore, in situ measurements were too labor intensive and subject to several experimental biases, hampering proper investigation of local adaptation in alpine plants. The recent availability of very high resolution (VHR) digital elevation models (DEMs) (<1 m) has made it possible to effectively approximate ecologically meaningful variables with limited fieldwork, offering the type of fine‐scale environmental data that are required to assess both the scale of adaptive patterns and the underlying factors in heterogeneous landscapes (Leempoel et al., 2015). DEM‐derived variables such as temperature, soil moisture, or solar radiation are easy to compute and have the potential to be widely used as proxies in ecology and evolution (Kozak, Graham, & Wiens, 2008; Leempoel et al., 2015; Wilson & Gallant, 2000). However, such topographic variables have rarely been used in landscape genetics and need to be further evaluated (Leempoel et al., 2017).

It is intuitively expected that the higher resolution of a DEM is likely to produce more accurate results, although it appears that a high amount of details may blur the output signal (Cavazzi, Corstanje, Mayr, Hannam, & Fealy, 2013). Studies in geomorphology have indeed shown that the relationship between DEM variables and physical characteristics of the terrain could only be valid at a specific spatial resolution (Kalbermatten, Van De Ville, Turberg, Tuia, & Joost, 2012; Wilson & Gallant, 2000). In contrast, research in landscape ecology has rarely considered the influence of the spatial resolution of environmental data. The relevance of topographic variables in species distribution models has been regularly reported (Le Roux, Virtanen, & Luoto, 2013; Lefsky, Cohen, Parker, & Harding, 2002; Randin, Vuissoz, Liston, Vittoz, & Guisan, 2009). However, some studies showed substantial improvement of models attributed to finer environmental variables (Camathias, Bergamini, Küchler, Stofer, & Baltensweiler, 2013), whereas others found limited differences (Pradervand, Dubuis, Pellissier, Guisan, & Randin, 2014). Noticeably, DEM‐derived variables have rarely been used in Gene‐Environment Associations and, to our knowledge, the spatial resolution has never been considered as an influencing parameter, which likely leads to incomplete conclusions on local adaptation (Manel, Poncet, Legendre, Gugerli, & Holderegger, 2010; Parisod & Joost, 2010; Storfer, Murphy, Spear, Holderegger, & Waits, 2010).

In this study, we explored the population structure of the alpine plant *Biscutella laevigata* and performed correlations between local environmental data and genetic variation. To do so, we used 233 polymorphic AFLP markers and 13 VHR DEM‐derived variables, demonstrated as relevant environmental proxies (Leempoel et al., 2015). Our aims were to (i) detect fine‐scale population structure, (ii) evaluate to what extent DEM‐derived proxies of environmental features are powerful to detect signatures of selection, (iii) assess the impact of their spatial resolution on the detection of signatures of selection. Taking advantage of very high resolution, we thus here appraise and discuss the scale dependency of microhabitat modeling and of signatures of selection.

## 2 MATERIAL AND METHODS

### 2.1 Sampling

*Biscutella laevigata* is a widespread polyploid Brassicaceae species that occurs mostly as small patches across the European Alps (Parisod & Besnard, 2007). This strictly outcrossing, perennial plant has its pollen dispersed by generalist Diptera and Lepidoptera, while seeds disperse through gravity and possibly wind (Parisod & Bonvin, 2008).

The study zone is situated at “les Rochers‐de‐Naye” (N46°26′00″, E6°58′50″), where a natural hybrid zone between closely related *B*. *laevigata* lineages has been documented along a 1.2‐km‐long ridge at an elevation included between 1,864 and 2,043 m above sea level (Parisod & Christin, 2008). Across the whole populated area, 361 individuals of *B. laevigata* were selected using a random cluster sampling strategy to represent the spatial distribution of the population. Selected areas of 4 × 4 m, separated by random distances of 0 to 25 m, were subdivided in four 2 × 2 m plots that were sampled when at least five individual plants were present. If less than five individuals were found in any of the four plots, a new area, at least 25 m further along the ridge, was selected. All individuals where georeferenced using a differential GPS offering a horizontal accuracy of c. 2–3 cm and a vertical accuracy of c. 3–4 cm. Their leaves were immediately dried in silica gel for extraction of genomic DNA following a standard DNeasy plant extraction mini kit protocol from Qiagen AG, Switzerland.

### 2.2 AFLP genotyping, scoring, and error estimation

All individuals were genotyped with amplified fragment length polymorphisms (AFLPs) following Parisod and Christin (2008). Despite limitations inherent to their dominant nature, AFLP loci are widely distributed across the genome and support appropriate genotyping that is hardly outperformed by current high‐throughput approaches in polyploids (Mason, 2015). In short, genomic DNA was digested with *Eco*RI and *Mse*I before ligation of adaptors to perform preselective and selective amplifications. PCR products amplified with FAM, VIC, NED fluorescent dye on the *Eco*RI primers were pooled with GeneScan 500 LIZ Size ladder and separated the 3730xl DNA analyzer capillary sequencer (Applied Biosystems). Resulting electropherograms were scored between 75 and 500 bp with GENEMAPPER v. 4.0 (Applied Biosystems) using AFLP default peak detection parameters. The scoring was checked manually, and AFLP loci were recorded as present (1) or absent (0) in binary matrices.

After an initial assessment of polymorphism and reproducibility of 38 AFLP primer combinations, the six bests (MCAG/EATC, EAGG/MCGG, MCAG/EAAT, EACT/MCAC, MCGA/EATA, and MCGG/EATA) were retained for genotyping. Individuals were randomly distributed among plates and the whole procedure was replicated on 15% of the samples to evaluate the error rate sensu Bonin, Taberlet, Miaud, and Pompanon (2006).

### 2.3 Population structure and gene flow

An issue regularly encountered when studying patterns of genetic variation and local adaptation in plant populations is recent polyploidy (Meyers & Levin, 2006). As polyploid populations strongly violate Hardy–Weinberg expectations, most standard methods in population genetics cannot be applied (Ronfort, Jenczewski, Bataillon, & Rousset, 1998). Furthermore, inferential frameworks accounting for the evolutionary genetics of polyploids must rely on accurate datasets assessing dosage of the multiple alleles at each locus, which still is technically challenging with high‐throughput genotyping (Mason, 2015). Accordingly, approaches deprived from population genetics pre‐requisites should currently be privileged among the applicable methods to evaluate local adaptation from genetic data. Under such circumstances, the population structure can profitably be described using, for example, the K‐mean clustering or principal component analysis, whereas the detection of signatures of selection can be achieved using generalized regressions or mixed models (Parisod & Joost, 2010).

Unbiased inference of population genetic structure was here assessed using K‐means clustering, a data partitioning method implemented in the R package Vegan (Dixon, 2003), with the Calinski criterion (Caliński & Harabasz, 2007) to select the most likely number of genetic clusters (Gompert, Lucas, Fordyce, Forister, & Nice, 2010). Accordingly, individual AFLP genotypes were assigned to their genetic cluster using the fuzzy c‐means algorithm (Dunn, 1974) implemented in the package “e1071” in R (Meyer, Dimitriadou, Hornik, Leisch, & Weingessel, 2014) with fuzzification parameter optimized at 1.02. Such a genomic cline approach was successfully used to dissect gene flow between polyploid taxa across natural hybrid zones (e.g., Senerchia et al., 2016). After a maximum of 1,000,000 iterations, the outputs of 1,000 independent runs of this algorithm were combined in CLUMPP (Jakobsson & Rosenberg, 2007) using the Greedy search method and 10,000 repeats of random input order. The coefficient of membership to a cluster was provided by CLUMPP as the mean of the independent runs. Such an individual coefficient can be considered as an admixture score estimated without biological assumptions. Accordingly, individuals were considered as belonging to population A and population B when their coefficient of membership was below 0.2 and above 0.8, respectively. Individuals with intermediate scores were considered as admixed.

Spatial genetic structure was quantified using SPAGeDi (Hardy & Vekemans, 2002), which measures the pairwise relatedness between individuals at increasing distance intervals. The mean relationship coefficient among loci was computed within 20 balanced intervals (i.e., with the same number of pairwise comparisons in each interval), and its significance was assessed with 9,999 permutations between individuals.

### 2.4 Environmental variables

The acquisition of the VHR DEM used in this study is described into details in Leempoel et al. (2015). Briefly, a LIDAR point cloud was obtained over the study area (helicopter) and transformed into a DEM with a resolution of 0.5 m. The following 13 variables were then derived from this DEM using SAGA GIS (Conrad et al., 2015): northness (Cosine of Aspect, Nor), eastness (Sine of Aspect, Eas), slope, vector ruggedness measure (VRM) (Sappington, Longshore, & Thompson, 2007), total solar radiation in June and December (Ti6, Ti12) (Böhner & Antonić, 2009), positive and negative topographic openness (TOP and TON) (Yokoyama, Shirasawa, & Pike, 2002), sky view factor (SVF) (Häntzschel, Goldberg, & Bernhofer, 2005), wetness index (SWI) (Beven & Kirkby, 1979), flow path length (FPL) (O'Callaghan & Mark, 1984), and wind exposure index (WEX) (Conrad et al., 2015). VRM is a measure for the unevenness of terrain and distinguishes between rocky vs. smooth terrain. TOP and TON express the protection of a focal point from the surrounding relief. It is based on the maximum angle found at zenith (TOP) or at nadir (TON) from the point, over a defined radius. SVF expresses the ratio of the radiation received by a planar surface over the radiation emitted by the entire hemispheric environment. SWI is the logarithm of the ratio between the catchment area and the tangent of slope and quantifies the topographic control of hydrological processes. FPL calculates the upstream or downstream distance along the flow path for each sample. More details can be found in Leempoel et al. (2015).

In order to account for the variability of DEM‐derived variables due to spatial resolution in association models, each variable was computed at 0.5‐, 1‐, 2‐, 4‐, and 8‐m resolution by downgrading the original VHR DEM at coarser resolutions using a B‐spline filter (Kalbermatten et al., 2012), implemented in MATLAB (MATLAB Version 12b. Natick, MA, USA: The MathWorks Inc., 2010).

The values of the different DEM‐derived variables were compared between the three groups corresponding to populations A, B, and admixed individuals (see Results) with Kruskal–Wallis tests performed in R.

### 2.5 Detection of outlier loci

Association models between the presence of genetic markers and the value of DEM‐derived variables were processed using generalized linear mixed models (GLMMs) (Bolker et al., 2009; Zuur, Ieno, Walker, Saveliev, & Smith, 2009), which are advantageously independent of any genetic model. GLMMs are used in Gene‐Environment Associations studies to account for pseudo‐replication due to population structure among samples. In our case, we considered the pixel (i.e., each unit of the DEM grid) to be the random effect instead of the sampling plot or the genetic subpopulation. In fact, with DEMs at different resolutions, individuals often fall within the same pixel (i.e., should be considered as pseudo‐replicates) and we therefore used pixels’ IDs as the random parameter at each spatial resolution. The coarser the resolution, the more samples are located in the same pixel. Hence, samples are present in 295 pixels at a resolution of 0.5 m, 227 at 1 m, 140 at 2 m, 99 at 4 m, and 66 at 8 m. GLMMs were performed using the R package lme4 (Bates & Maechler, 2009) between each polymorphic marker and DEM variables using a binomial link function. Significance of all associations was assessed with a log‐likelihood ratio test, and AICs were compared between a model with a variable and a constant model. In addition to these variables, GLMM models were also performed with measured altitude (Alt), longitude (X), latitude (Y), as well as membership coefficient to population A. In these cases, the plot was considered as the random effect.

## 3 RESULTS

### 3.1 Population structure and gene flow

**Figure 1:**
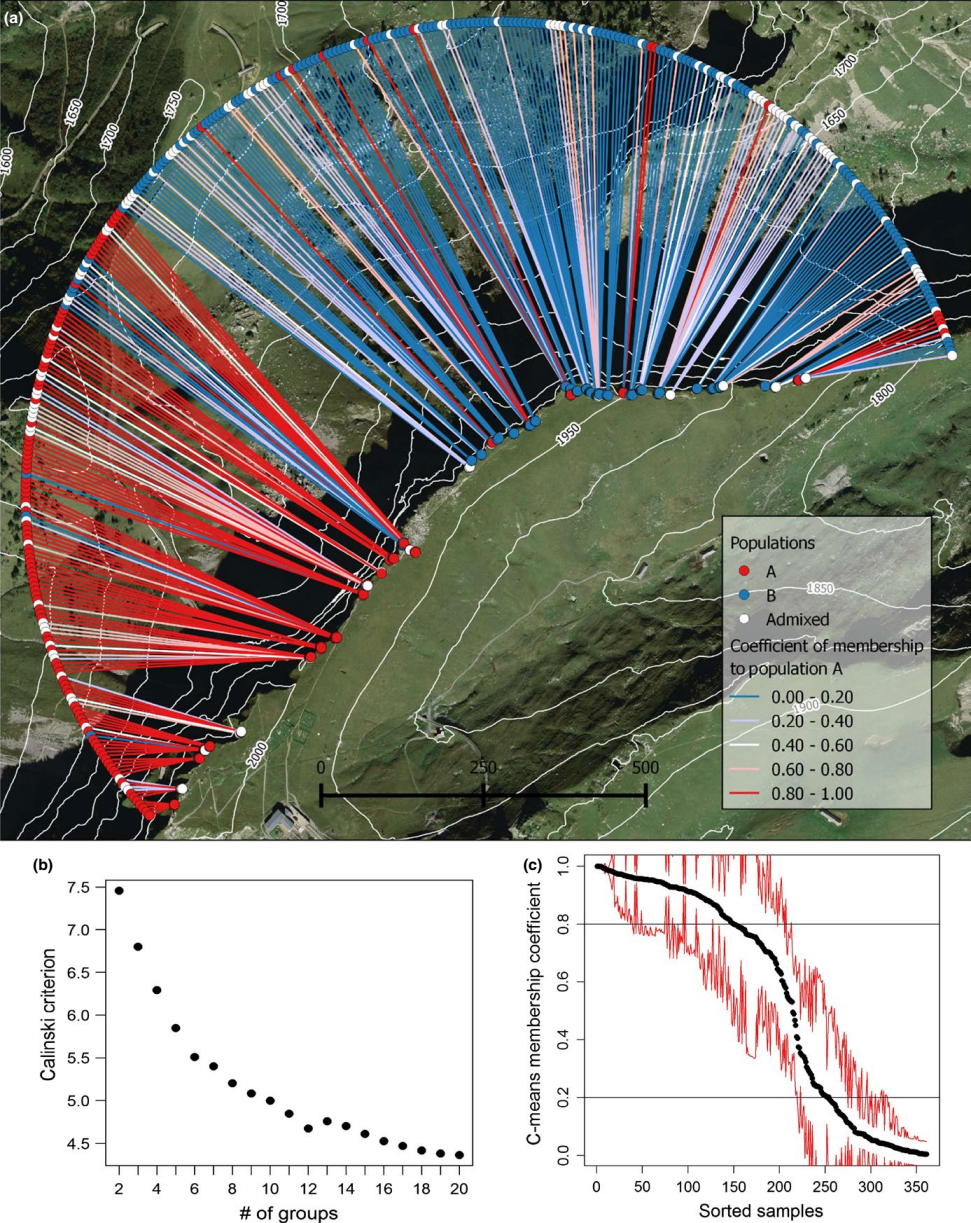
Population structure of the studied individuals. (a) Coefficients of membership to Population A obtained from C‐mean clustering are shown in (a) for each individual along the ridge. A semi‐circle was added to facilitate the visualization of the coefficients. (b) The Calinski Criterion values for the K‐mean clustering from 2 to 20 populations indicate that the most likely number of populations is 2. Finally, (c) shows the sorted membership coefficient to Population A and the standard error for each individual over the 1,000 iterations of the C‐means clustering, combined in Clumpp

Genotyping of the 361 individuals of *B. laevigata* yielded 233 polymorphic AFLP loci (frequency of minor variant >0.05) with an error rate estimated at 2.93% based on the replication of 15% samples. Clustering approaches evaluated the genetic structure based on individual genotypes, with the Calinsky criterion of the K‐means method highlighting two main genetic clusters within the study area (Figure 1b). Accordingly, C‐means clustering was performed for *K* = 2 over 1,000 iterations that were then assembled in Clumpp (Average pairwise similarity among replicates is 0.81). A majority of the individuals were unambiguously assigned to each of these genetically homogeneous clusters, whereas 105 admixed genotypes presented coefficients of membership between 0.2 and 0.8 (Figure 1c). Standard deviation over 1,000 runs was also relatively high for most of these admixed individuals.

**Figure 2:**
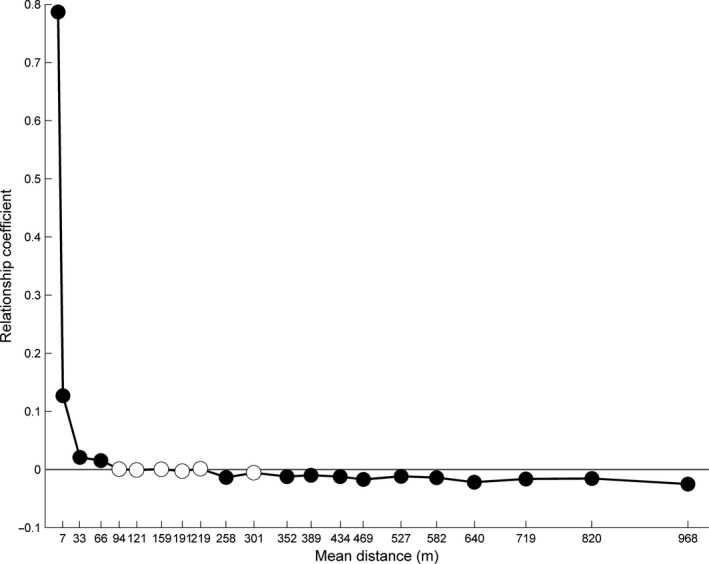
Pairwise relationship coefficient for AFLP markers in *Biscutella laevigata*. Pairwise relationships are calculated for 20 intervals of distances and are shown in black when significant (*p*‐value <.05/20) and in white otherwise

The spatial distribution of these genetic clusters (i.e., populations) showed a clear segregation (Figure 1a). Genotypes with membership higher than 0.8 (i.e., population A; 107 individuals) were mostly located on the upper part of the ridge, separated from population B (i.e., genotypes with membership lower than 0.2; 149 individuals) by a rocky area with very few individuals. Admixed individuals were reported across the whole area, with a slight bias toward the zone where population B is located. Despite such evidence of long‐range gene dispersal across habitats in this 1.2‐km‐long population, gene flow appeared consistently limited. Pairwise genetic relationship among individuals indeed declined considerably at short distances, after the second distance class (i.e., 7 m), and reached nonsignificant values from the fourth distance class (i.e., after only 66 m) (Figure 2).

### 3.2 Habitat comparison

All DEM‐derived variables, except TON, were found to be significantly different between populations (Table 1). For instance, population A was found to be located at a higher altitude than Population B. Population A was also more exposed to wind (WEX), presented a lower protection from surrounding relief (i.e., higher openness, TOP), a higher sky view proportion (SVF), and higher Terrain ruggedness (VRM) than population B. Interestingly, population A receives less solar radiation than B in December, but more in June. Regarding hydrology, individuals from population A were reported on significantly shorter flow path lengths (FPL) but showed higher soil wetness (SWI). Nevertheless, it is worth noting that some variables were only different among populations at particular resolutions. For example, Eas was significantly higher in Pop A than in Pop B at resolutions of 0.5, 1, 2, 4, and 8 m, while VRM was only higher in Pop A at 0.5 m.

**Table 1:** Comparison of DEM‐derived variables between habitats

Variable	Habitat A	Habitat B	Habitat admixed	Pvalue AB	Significant resolutions
Alt	1,994 (±33)	1,955 (±32)	1,964 (±37)	1.92E‐27	
Nor	−0.39 (±0.43)	−0.57 (±0.48)	−0.48 (±0.44)	3.70E‐10	0.5, 1
Eas	−0.05 (±0.65)	0.4 (±0.52)	0.31 (±0.62)	1.54E‐08	0.5, 1, 4, 8
Slo	34.661 (±18.751)	44.28 (±16.963)	47.564 (±17.793)	1.46E‐05	8
VRM	0.082 (±0.046)	0.068 (±0.053)	0.068 (±0.05)	9.96E‐05	0.5
TOP	1.472 (±0.1)	1.411 (±0.08)	1.434 (±0.101)	1.08E‐06	1
WEX	1.268 (±0.023)	1.257 (±0.021)	1.257 (±0.024)	1.21E‐06	0.5, 1, 2
SVF	0.8 (±0.1)	0.8 (±0.1)	0.7 (±0.1)	3.12E‐06	1, 8
Ti6	206.099 (±59.745)	169.79 (±70.372)	162.939 (±64.248)	4.07E‐05	8
Ti12	74.7 (±20.776)	86.121 (±21.817)	80.085 (±21.162)	4.38E‐10	0.5, 1, 8
FPL	27.29 (±37.08)	41.6 (±39.08)	41.55 (±42.04)	7.02E‐06	4
SWI	4.9 (±0.7)	4.5 (±0.7)	4.4 (±0.7)	2.18E‐05	8

Variables showing a significant difference between individuals of populations A, B and admixed ones are shown in the table. The mean and the standard deviation are given for each habitat. The following column provides the *p*‐value for the most significant Kruskal–Wallis test performed between Habitat A and B, and the final column indicates the resolutions at which the test was significant (<0.05 after Bonferroni's correction for multiple tests), that is, the variable in question is significantly different between the two populations at the spatial resolution indicated. Variables acronyms: altitude (Alt), northness (Nor), eastness (Eas), slope (Slo), vector rudggedness measure (VRM), positive topographic openness (TOP), wind exposure index (WEX), sky view factor (SVF), total insolation in June (Ti6), total insolation in December (Ti12), flow path length (FPL), SAGA wetness index (SWI). Pixel resolution is expressed in meters. DEM, digital elevation model

### 3.3 Detection of outliers

**Figure 3:**
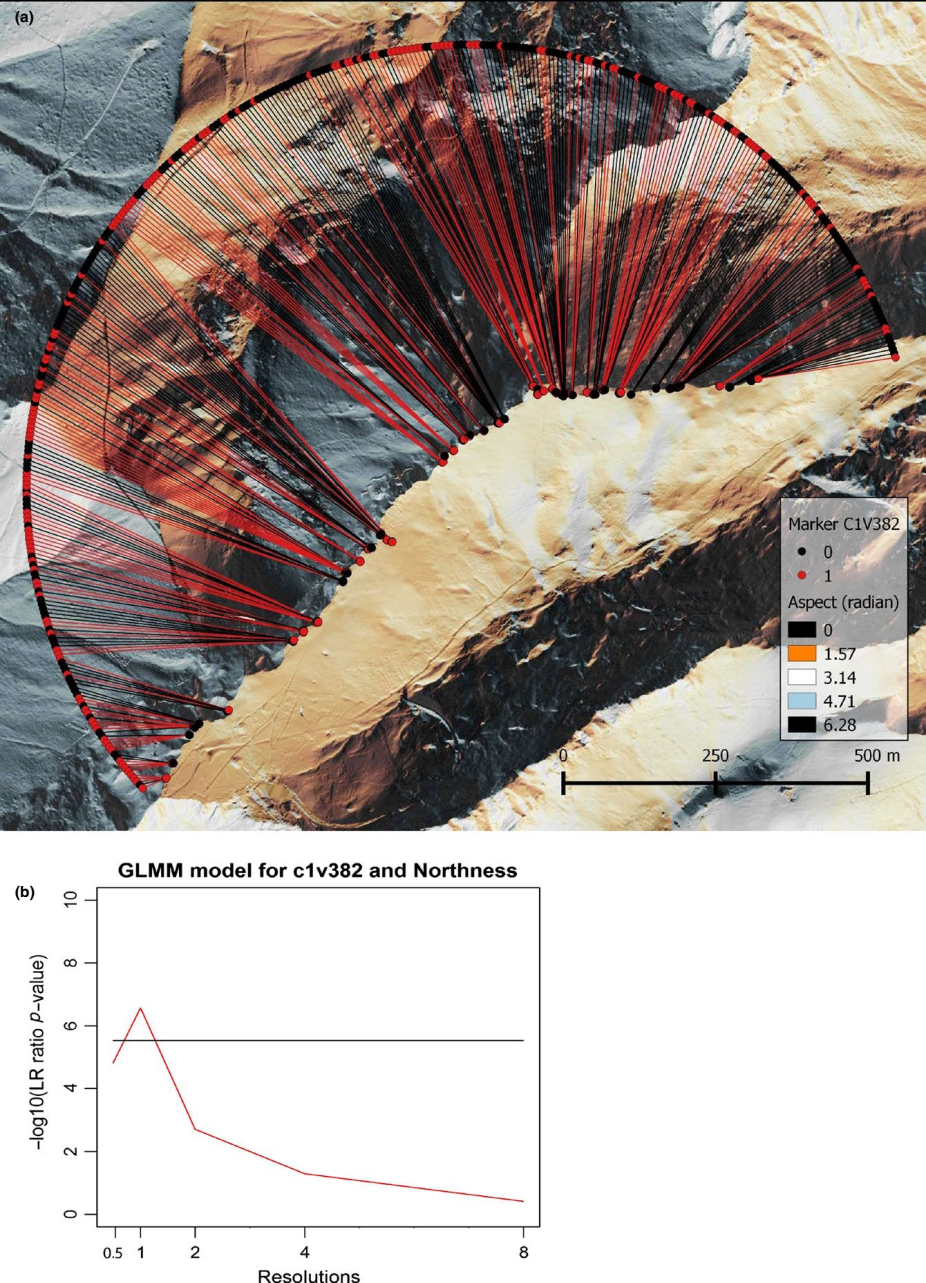
Variation of the significance of association models between genetic marker c1v382 and northness for different spatial resolutions. The distribution of the marker along the ridge is shown in a). The background represents the aspect computed at a resolution of 1 m. (b) shows the significance of GLMM with increasing resolution (i.e., pixel size of the DEM in meters), represented by the log10 of the *p*‐value of the log‐likelihood ratio. The horizontal bar is the significance threshold of 0.05 after Bonferroni's correction

Few GLMMs between genetic loci and environmental variables turned out to be significant with α = 0.05 after Bonferroni's correction (significance level: 3.3E‐06). Only five genetic markers were significantly associated with Alt, TON, Nor, Ti12, and WEX (Table 2). The spatial resolution of DEM‐derived variables, however, had a strong influence on the significance of associations. Indeed, these associations were only significant at a specific resolution. Although characterization of fine‐scale environmental heterogeneity appeared crucial, the highest resolution did not necessarily imply the highest significance. For example, the association between the locus c1v382 and Ti12 or Nor was only significant at 1 m and poorer at other resolutions (Figure 3). The two other associations, that is, c1v222 with TON and c1b136 with WEX, are only significant at the highest resolution but the former decreases more sharply at 1 m (see Appendix 1). Noticeably, none of the 233 loci were significantly associated with latitude, longitude, and membership coefficient to population A.

**Table 2:** Significant GLMM models as measured with the log‐likelihood ratio

Marker	Variable	Resolution	Likelihood ratio *p*‐value	β0	β1	AIC constant model	AIC variable model	Markers frequencies		
								Pop A	Pop B	admixed
c1v492	Alt		7.24E‐16	−9.16	−0.45	453.4	390.3	0.25	0.36	0.37
c1v222	TON	0.5	1.16E‐14	−9.10	−0.47	469.9	412.3	0.31	0.42	0.38
c1v382	Nor	1	2.75E‐07	−0.19	0.72	494.1	469.6	0.51	0.44	0.42
c1v382	Ti12	1	3.75E‐07	−0.20	−0.71	494.1	470.2	0.51	0.44	0.42
c1b376	Alt		5.91E‐07	−1.89	1.12	358.3	335.4	0.33	0.15	0.18
c1b136	WEX	0.5	1.85E‐06	−0.53	5.61	491.4	470.7	0.51	0.45	0.50

*p*‐Value, regression coefficients (β0 and β1), and AICs are provided for each model as well as the frequency of the genetic markers in each population. Both the AIC of the constant model and the AIC of the model including the variable are provided. Variables acronyms: altitude (Alt), negative topographic openness (TON), northness (Nor), total insolation in December (Ti12), wind exposure index (WEX). Pixel resolution is expressed in meters. GLMM, generalized linear mixed model

## 4 DISCUSSION

The fine‐scale environmental heterogeneity of mountainous regions makes such landscapes ideal for the study of patterns of local adaptation. In this research, we report evidence of local differentiation and signatures of selection in an alpine plant population of *B. laevigata*. We indeed evidenced a marked spatial genetic structure along a 1.2‐km ridge on which two coherent gene pools were identified, separated by an unsuitable rocky area (Figure 1a). Consistent with a neutral pattern under restricted gene flow, genetic similarity between neighboring individuals declined abruptly and appeared nonsignificant after 64 m only. This result exhibits a particularly high isolation‐by‐distance in the continuously sampled population over the studied ridge. However, admixed individuals were highlighted all over the studied area, indicating that homogenizing gene flow is likely at work across the population. The habitat of individuals genetically assigned to these two populations showed substantial differences in local topographic conditions and we further report signatures of selection on specific loci due to local environmental factors. Those results are consistent with the mosaic distribution of subalpine and alpine lineages reported along a regular transect at this ridge (Parisod & Christin, 2008). Among the five genetic markers here strongly associated with DEM variables, one candidate adaptive locus was associated with solar radiation in December and northness and is thus further congruent with prior results suggesting selection by solar radiation (i.e., degree‐days during the growing season and total solar radiation) as evidenced by Parisod and Joost (2010).

Those results do not unambiguously support local adaptation as the unoccupied rocky area may act as a strong barrier limiting gene flow. Consequently, it is not unlikely that genetic drift acted on both populations independently and that the influence of the clear habitat demarcation was not the major driver of the reported genetic differentiation. Accordingly, we found only a limited number of significant associations, despite the substantial number of environmental variables tested and the high number of individuals sampled. In contrast to predictions of such neutral model ruled by demography, the reported pattern also matched with expectations under weak selection, with the spatial distribution of significant associations along environmental gradients only weakly reflecting the population structure, and thus making them plausible signatures of selection. The observation that none of the identified genetic markers is correlated with the population membership coefficient is further consistent with adaptive processes having shaped the distribution of slightly more than 2% of the loci being surveyed.

Bearing limitations of the approach in mind, our observations demonstrate that VHR DEMs can be suitably used to model fine‐scale environmental heterogeneity. Among investigated topographic variables, solar radiation, terrain ruggedness, and wind exposure appear to substantially differ between the two populations. Noticeably, they reflect climatic variability at microsite that is only identified by fine‐scale topographic models, demonstrating their usefulness for landscape genomics studies requiring such resolution (Leempoel et al., 2015; Manel et al., 2010; Pradervand et al., 2014). Most importantly, the DEM‐derived variables used here were shown to be surrogates for relevant ecological features, including temperature and snow cover variability in mountainous areas (Böhner & Antonić, 2009; Leempoel et al., 2015; Lehning, Grünewald, & Schirmer, 2011; Wilson & Gallant, 2000). Accordingly, we show here that a large panel of variables exist and can likely be expanded to refine environmental characterization for many organisms. For instance, vector ruggedness measure (VRM) appeared to be the most important predictor of soil moisture on the ridge (Leempoel et al., 2015). High‐resolution VRM thus appears as a suitable proxy for the distribution of stony areas and more generally soils with different porosities. Exposure to wind was also noticeable in habitat comparisons and in association models. As it indirectly affects snow accumulation (Plattner, Braun, & Brenning, 2004) and thus the timing of snow removal in alpine habitats, which we observed to be correspondingly heterogeneous over the study site (pers. obs.), wind exposure represents a useful proxy for gathering insights on the start of the growing season or exposure to cold during the harsh season. In addition, altitude has an important role as two markers identified of six were associated with this variable only. Clearly, it remains among the most important parameter influencing temperature at any scale in mountainous regions (Leempoel et al., 2015; Wilson & Gallant, 2000).

Multiscale models used here enabled precise analyses, thanks to ecologically relevant topographic proxies (Leempoel et al., 2015). For both habitat comparisons and association models, we report a high sensitivity to spatial resolutions and a generally decreased strength of GLMM models at coarser resolutions, which were mostly nonsignificant. It appears that DEM‐derived variables computed at a single resolution, particularly at coarse ones, do not fully represent the topographic control on ecologically relevant variables, and are not able to replicate at best the spatial continuum naturally constituting landscapes. Noticeably, associations between genetic markers and different environmental variables did not generally converge toward an optimal resolution, indicating that the suitable resolution depends on the type of DEM‐derived variable considered (Leempoel et al., 2015).

Our framework illustrates that ecologically relevant DEM‐derived proxies are relatively easy to acquire and provide unique information on micro‐habitats for landscape genomics studies. However, we also highlight their sensitivity to changes in spatial resolution and argue that the interpretation of results obtained from DEMs at a single resolution should be cautiously considered. By no means, a single resolution, even the finest, may be sufficient to identify signatures of selection in highly heterogeneous landscapes. Accordingly, recommending an appropriate scale would likely be misleading and we rather suggest that future studies be based on high‐resolution models to explore multiscale derived variables, as we did in this study. While we focused on a single species, we expect these recommendations to be valid for a broad range of taxa and habitats. On the other hand, coarse resolution climatic variables interpolated over homogeneous landscapes may be sufficient for specific situations (Fick & Hijmans, 2017) that are unlikely to benefit from a multiscale approach.

## DATA ACCESSIBILITY

Genetic and Environmental data are available on Zenodo.

## AUTHORS CONTRIBUTIONS

KL conducted fieldwork, performed analysis, and wrote the manuscript. CP designed and supervised the project and contributed to the writing. CG conducted fieldwork, performed the genetic analysis, and contributed to the discussion. SJ supervised the project and contributed to the writing.
